# Thank you to *Virology Journal*’s peer reviewers in 2012

**DOI:** 10.1186/1743-422X-10-44

**Published:** 2013-02-20

**Authors:** Linfa Wang

**Affiliations:** 1 CSIRO Animal Food and Health Sciences, Australian Animal Health Laboratory, Geelong, VIC, 3220, Australia; 2 Program in Emerging Infectious Diseases, Duke-NUS Graduate Medical School, Singapore, 169857, Singapore

## Abstract

**Contributing reviewers:**

The editors of *Virology Journal* would like to thank all our reviewers who have contributed to the journal in Volume 9 (2012). The success of any scientific journal depends on an effective and strict peer review process and *Virology Journal* could not operate without your contribution. We look forward to your continuous support to this journal either as an invited reviewer or a contributing author in the years to come.

## 

John Aaskov

Australia

Dharam Ablashi

USA

Celia Abolnik

South Africa

Sazaly AbuBakar

Malaysia

Ishag Adam

Sudan

Stuart Adler

USA

Claudio Afonso

USA

Alessio Aghemo

Italy

Patricia Aguilar

USA

Simon Agwale

Nigeria

Kamruddin Ahmed

Japan

Insung Ahn

South Korea

Wataru Akahata

USA

Norio Akuta

Japan

Seyed-Moayed Alavian

Iran

George Alemnji

USA

Mubarak Alfaresi

United Arab Emirates

Gabriel Almeida

Brazil

Xavier Alvarez

USA

Roberto Alvarez-Lafuente

Spain

Angela Amedee

USA

Samad Amini-Bavil-Olyaee

USA

Alongkorn Amonsin

Thailand

Dong-Jun An

South Korea

Angelo Andriulli

Italy

German Añez

USA

Gioacchino Angarano

Italy

Victor H Aquino

Brazil

Abdelsatar Arafa

Egypt

Natalia Araujo

Brazil

Joselio Araujo

Brazil

Juan Arbiza

Uruguay

Patrick Arbuthnot

South Africa

Carlos Arias

Mexico

Luciana Arruda

Brazil

Trino Ascencio-Ibanez

USA

Usman ali Ashfaq

Pakistan

Susana Asin

USA

Houssam Attoui

UK

Walter Atwood

USA

Prasert Auewarkul

Thailand

Laure Aurelian

USA

Sita Awasthi

USA

Sina Aziz

Pakistan

Shawn Babiuk

Canada

Malin Backstrom

Sweden

Olfa Bahri

Tunisia

Lang Bai

China

Andrea Baier

Poland

Saeeda Baig

Pakistan

Udeni Balasuriya

USA

Fausto Baldanti

Italy

Dennis Bamford

Finland

Bruce Banfield

Canada

Anju Bansal

India

Krisztian Banyai

Hungary

Lihua Bao

USA

Veronique Barban

France

Dale Barnard

USA

Michael Baron

UK

Ian Barr

Australia

Alan Barrett

USA

Noel Barrett

Austria

Naina Barretto

USA

Birke Bartosch

France

Saroj Basak

USA

Armanda Bastos

South Africa

Daniel Bausch

USA

Aysen Bayram

Turkey

David Beasley

USA

Maria Serena Beato

Italy

J. David Beckham

USA

Nargis Begum

India

Abbas Behzad Behbahani

Iran

Mark Beilke

USA

Jerome Belinson

USA

Nancy Bellei

Brazil

Gael Belliot

France

Carla María Bellomo

Argentina

Dirk Bellstedt

South Africa

Robert Belshaw

UK

Reinout Alexander Bem

Netherlands

David Benfield

USA

Abdelouaheb Bennani

Morocco

Jeffrey Bergelson

USA

Alan Berger

Canada

Sven M. Bergmann

Germany

Ben Berkhout

Netherlands

Jesus F. Bermejo-Martin

Spain

Philip Berry

UK

Jonathan Bertin

Canada

Mael Bessaud

France

Walter Betancourt

Venezuela

V. Bhanuprakash

India

Purnima Bhat

Australia

Neerja Bhatla

India

Emiliano Biasini

USA

Mirna Biglione

Argentina

Marco Binder

Germany

Achilea Bittencourt

Brazil

Stuart Blacksell

Thailand

Carol Blair

USA

Esther Blanco

Spain

Joe Blaney

USA

Andrew Blann

UK

Evan Bloch

USA

Sandra Blome

Germany

David Bloom

USA

Amy Blum

Canada

Enrique Boccardo

Brazil

Thomas Bock

Germany

Myrna Bonaldo

Brazil

Jacco Boon

USA

David Booth

Australia

Didier Boquet

France

Durlav Prasad Bora

India

Kocjan Bostjan

Slovenia

Marcelle Bottecchia

Brazil

Jason Botten

USA

Lamjed Bouslama

Tunisia

David Boyle

Australia

A. Mithat Bozdayi

Turkey

Andrea Branch

USA

Luis Branco

USA

Curtis Brandt

USA

Catherine Brennan

USA

Rob Briddon

Pakistan

Emiliana Brocchi

Italy

Susan Brockmeier

USA

Shobha Broor

India

Lorena Brown

Australia

Jay Brown

USA

Earl Brown

Canada

Glenn Browning

Australia

Wolfram Brune

Germany

Volker Bruss

Germany

Michael Buchmeier

USA

Amy Buck

UK

Agata Budkowska

France

Alexander Bukreyev

USA

Rowena Bull

Australia

Franco Maria Buonaguro

Italy

Johan Burger

South Africa

Robert Burk

USA

Oscar Burrone

UK

Felicity Burt

South Africa

Michael Burwinkel

Germany

Ismael Bustos-Jaimes

Mexico

Daniel Cadar

Romania

Todd Callaway

USA

Amanda Calvert

USA

Edward Campbell

USA

Mary Campbell

USA

Corey L. Campbell

USA

Rodolfo Campos

Argentina

Daniel Candotti

UK

Edouard Cantin

USA

Yongchang Cao

China

Dianjun Cao

USA

Timothy Cardozo

USA

Vincenzo Carginale

Italy

M. Carr

Ireland

Jill Carr

Australia

Sandrine Castelain

France

Jaime Castellanos

Colombia

Jamie Cate

UK

Mattia Cecchinato

Italy

Avrelija Cencic

Slovenia

Valeria Cento

Italy

Escobedo Cesar

Belgium

Young Joo Cha

South Korea

Chanhee Chae

South Korea

Parin Chaivisuthangkura

Thailand

Supriya Chakraborty

India

Runu Chakravarty

India

Martin C.W. Chan

Hong Kong

Gary Chan

USA

Partha Chandra

USA

Huiyun Chang

China

Jinhong Chang

USA

Yung-Fu Chang

USA

Gwong-Jen Chang

USA

Mamta Chawla-Sarkar

India

Xiaoyan Che

China

Kow-Tong Chen

Taiwan

Zehua Chen

USA

En-Qiang Chen

China

Jiezhong Chen

Australia

Ze Chen

China

Xinwen Chen

China

Jian Chen

USA

Hualan Chen

China

Anchun Cheng

China

Yawen Cheng

Taiwan

Doo-Sung Cheon

South Korea

Peter Cherepanov

UK

Patricia Chevez-Barrios

USA

Angela Chi

USA

Glenys Chidlow

Australia

Sadegh Chinikar

Iran

Charles Chiu

USA

Kyoung-Oh Cho

South Korea

Kyoung-Seong Choi

South Korea

Steve Choi

USA

Young Ki Choi

South Korea

Kang-Seuk Choi

South Korea

Shafiqul Chowdhury

USA

Vauloup-Fellous Christelle

France

Urs Christen

Germany

Kaw Bing Chua

Singapore

Sun-Ku Chung

South Korea

Karen Coats

USA

Alan Cochrane

Canada

Don Coen

USA

Carla Coffin

Canada

J. Robert Coleman

USA

Martine Collumbien

UK

Margaret E. Conner

USA

Fiona Constable

Australia

Nigel Cook

UK

Anthony Corfield

UK

Adriana Correa

Brazil

Marti Cortey

France

Ömer Coskun

Turkey

Maria Isabel Costafreda

Spain

Therese Couderc

Gabon

Pierre Coursaget

France

Anthony Craig

USA

Maria Isabel Craig

Argentina

Randy Cron

USA

Maria Grazia Cusi

Italy

Flávio da Fonseca

Brazil

Jack da Silva

Australia

Paul D’Agostino

USA

Kaifan Dai

USA

Mohammed Arshud Dar

Canada

Indranil Dasgupta

India

Paban Kumar Dash

India

David Davido

USA

Irit Davidsoni

Israel

Salvatore Davino

Italy

Charles Davis

USA

Andrew Davison

UK

Maurits de Koning

Netherlands

Xavier de Lamballerie

France

Nicola Decaro

Italy

Sehgal Deepak

India

Mark Delboy

USA

Adriana Delfraro

Uruguay

Rafael Delgado

Spain

Qiang Deng

China

Jonathan Dennis

Canada

Cynthia Derdeyn

USA

Joe DeRisi

USA

Philippe Despres

France

Vincent Deubel

Cambodia

Tapan Dhole

India

Youxiang Diao

China

Russell Diefenbach

Australia

Richard Dix

USA

Thomas Dobner

Germany

Antonina Dolei

Italy

Stephen Dollery

USA

Joseph Domachowske

USA

Samuel Dominguez

USA

John Dong

USA

David Donovan

USA

Carlos P. Dopazo

Spain

Anton Dormer

USA

Jos Dortmans

Netherlands

Daniel Dory

France

Joseph Dougherty

USA

Trevor Drew

UK

Jan Drobeniuc

USA

Christian Drosten

Germany

Lanying Du

USA

Claudia Duarte dos Santos

Brazil

Robert Duda

USA

Joseph Dudley

USA

Santiago Dueñas-Carrera

Cuba

Carol Duffy

USA

Emanuele Durante Mangoni

Italy

Hideki Ebihara

USA

Kenneth Eckels

USA

Stacey Efstathiou

UK

Christina Ehrhardt

Germany

Damon Eisen

Australia

Damian Ekiert

USA

Mostafa El-Awady

Egypt

Nels Elde

USA

Deilson Elgui de Oliveira

Brazil

Gabriella Elia

Italy

Mortada El-Shabrawi

Egypt

Kerry Empey

USA

Otto Erlwein

UK

Susanna Esposito

Italy

Pedro Esteves

Portugal

Nicholas Eyre

Australia

Caio Fagundes

Ireland

DeLisa Fairweather

USA

Erik Falck-Pedersen

USA

Shufang Fan

USA

Hongjie Fan

China

Xue-Gong Fan

China

Ying Fang

USA

Weihuan Fang

China

Tibor Farkas

USA

Claude Fauquet

USA

Michel Favre

France

Dino Feigelstock

USA

Isabella Felli

Italy

Pascal Fender

France

Li Feng

China

Brian Ferguson

UK

Humberto Fernandes

UK

Luca Ferrari

Italy

Paula Ferreira

Portugal

Jaqueline Maria Siqueira Ferreira

Brazil

Luís Ferreira

Brazil

Ralph Feuer

USA

Jonathan Filee

France

William Finlay

Ireland

Gustavo Fioravanti Vieira

Brazil

Andrew Firth

UK

Nicole Fischer

Germany

Susan Fisher-Hoch

USA

Svetlana Folimonova

USA

Petra Forgách

Hungary

Naomi Forrester

USA

Puri Fortes

Spain

Caroline Fossum

Sweden

Ashley Fowlkes

USA

Nicole Frahm

USA

Lorenzo Fraile

Spain

Arthur Frampton

USA

Alan Franklin

USA

Conrad Freuling

Germany

David Frick

USA

Zhen Fu

USA

Marc Fuchs

USA

Mikiya Fujieda

Japan

A. Oveta Fuller

USA

Nicholas Funderburg

USA

James Fung

Hong Kong

Yuki Furuse

Japan

Alice Fusaro

Italy

Gülsah Gabriel

Germany

Liliana Gabrielli

Italy

Jose Paulo Gagliardi Leite

Brazil

Nicolas Gaidet

France

Stefania Galdiero

Italy

Monica Galiano

UK

Lilianne Ganges

Spain

Feng Gao

USA

David Garboczi

USA

Maria Luz Garcia

Spain

Gissel García

Cuba

Ramon Garcia-Escudero

Spain

Ana L. Garcia-Perez

Spain

Jeremy Garson

UK

Kyle Garver

Canada

Silvana Gaudieri

Australia

Brian Geiss

USA

Maria Magdalena Gherardi

Argentina

Homayon Ghiasi

USA

Reena Ghildyal

Australia

Sudip Ghosh

India

Souvik Ghosh

Japan

David Giedroc

USA

Lazaro Gil

Cuba

Sarah Gilbert

UK

Martin Gilbert

USA

Robert Gilbertson

USA

Sabine Gilch

USA

Michelle Giles

Australia

Jason Gill

USA

Anna Gillio-Tos

Italy

Miroslav Glasa

Slovakia

Joel Glasgow

USA

Peter Goff

USA

William Golde

USA

Selma de Andrade Gomes

Brazil

Jaime Gómez-Laguna

Spain

Fernando Gonzalez-Candelas

Spain

Steve Goodbourn

UK

Maureen Goodenow

USA

Andrew Goodwin

USA

Uri Gophna

Israel

Michelle Gordon

South Africa

David Gordon

Australia

Ernest Gould

UK

Ioannis Goulis

Greece

Elena Govorkova

USA

Sheila Graham

UK

Simon Graham

UK

Marc Grandadam

France

Beatrice Grasland

France

Alberto Grassi

Italy

Cecilia Graziosi

Switzerland

Laura Green

UK

Harry Greenberg

USA

David Gretch

USA

Thomas Grunewald

Germany

Hongxi Gu

China

Rodrigo Guabiraba

UK

Jiewen Guan

Canada

Severin Gudima

USA

Jerome Guechot

France

Jean-Luc Guerin

France

Gilles Guillemin

Australia

Susana Guix

Spain

Hailong Guo

USA

Haitao Guo

USA

Peng Guoping

China

Klaus Gutfreund

Canada

Bernd Haas

Germany

Mojgan Haddad

USA

Steohanos Hadziyannis

Greece

Michael Hagensee

USA

Susan Hahne

Netherlands

Benjamin Hale

UK

Kim Halpin

Singapore

Eric Halsey

Peru

Scott Halstead

USA

Junzo Hamanishi

Japan

Klaus Hamprecht

Germany

Jun Han

USA

Ken-Ichi Hanaki

Japan

Kathryn Hanley

USA

Naila Hannachi

Tunisia

Wanna Hanshaoworakul

Thailand

Steve Hanson

USA

Olivier Hantz

France

Chanditha Hapuarachchi

Sri Lanka

Timm Harder

Germany

Andrew Harman

Australia

Robert Harrison

USA

Charlotte Hársi

Brazil

Heli Harvala

UK

Colin Harwood

UK

Futoshi Hasebe

Japan

Rie Hasebe

Japan

Syed Hashsham

USA

Todd Hatchette

Canada

Andrea Hawe

Germany

Michael Hawkes

Canada

Daisuke Hayasaka

Japan

Kazuhiko Hayashi

Japan

David Hayman

USA

Klaus Hedman

Finland

Regine Heilbronn

Germany

Michael Heinrich

UK

Zdenek Hel

USA

Ekaterina Heldwein

USA

Andreas Henke

Germany

Kathleen Henshilwood

Ireland

Georges Herbein

France

Roger Hewson

UK

Eberhard Hildt

Germany

Julia Hilliard

USA

Dirk Hoeper

Germany

Bernd Hoffmann

Germany

Ursula Hofle

Spain

Martin Hofmann

Switzerland

Regina Hofmann-Lehmann

Switzerland

Oliver Hohn

Germany

Mike Holbrook

USA

Kathryn Holmes

USA

Michael Holzel

Germany

Qiu Hongling

China

Nildimar Honório

Brazil

Jim-Tong Horng

Taiwan

Veit Hornung

Germany

M. Jahangir Hossain

Gambia

Weihong Hou

USA

Jinlin Hou

China

Wei-Li Hsu

Taiwan

Jianhe Hu

China

Yu-Chen Hu

Taiwan

Rongliang Hu

China

Zhihong Hu

China

Wei-Pang Huang

Taiwan

Mingjun Huang

USA

Claire Huang

USA

Karsten Hueffer

USA

Birgit Huelseweh

Germany

John Hughes

UK

Joseph Hughes

UK

Veijo Hukkanen

Finland

Jens Humrich

Germany

Chao-Hung Hung

Taiwan

Tahziba Hussain

India

Snawar Hussain

USA

Gero Hutter

Germany

Kathryn Huyvaert

USA

Eung Soo Hwang

South Korea

Hiroshi Ichimura

Japan

Masanori Ikeda

Japan

Kunitoshi Imai

Japan

Shinsaku Imashuku

Japan

Fumio Imazeki

Japan

Allison Imrie

Australia

Wataru Ishii

Japan

Tatsuo Ito

USA

Miren Iturriza

UK

Alexander Ivanov

Russia

Masako Iwanaga

Japan

Yoshiaki Iwasaki

Japan

Peter Jahrling

Afghanistan

Pooja Jain

USA

Kuan-Teh Jeang

USA

Victoria Jensen

USA

Holger Jeske

Germany

Renyong Jia

China

Ming-De Jiang

China

Ping Jiang

China

Chen Jianjun

China

Xia Jin

USA

F. Brent Johnson

USA

Nicholas Johnson

UK

Kathryn Jones

USA

Clinton Jones

USA

Marcel Jonges

Netherlands

Elsa Jourdain

France

Yong-Tae Jung

South Korea

Kwonil Jung

USA

Prapan Jutavijittum

Thailand

Tal Kafri

USA

Yoshihiro Kaku

Japan

John Kalbfleisch

USA

Johnan Kaleeba

USA

Amine Kamen

Canada

Kurt Kamrud

USA

Tatsuo Kanda

Japan

Hisatoshi Kaneko

Japan

Sang-Moo Kang

USA

Xiaoping Kang

China

Phyllis Kanki

USA

Rajesh Kannangai

India

Jia-Horng Kao

Taiwan

Amit Kapoor

USA

Stylianos Karatapanis

Greece

Peter Karayiannis

UK

Adam Karpala

Australia

Fatah Kashanchi

USA

Benedikt Kaufer

Germany

Johannes Kauffold

Germany

Nikolai Kaverin

Russia

Mark Kay

USA

Mirdad Kazanji

Central African Republic

Marcus Kehrli

USA

Alyson Kelvin

Canada

Shannon Kenney

USA

Daniel Kern

USA

Aurora Kerscher

USA

Madhu Khanna

India

Sunil Khattar

USA

Yury Khudyakov

USA

Wansika Kiatpathomchai

Thailand

Frederick Kibenge

Canada

Hiroshi Kida

Japan

Hiroshi Kido

Japan

Kwangpyo Kim

South Korea

Hye-Ryoung Kim

South Korea

Kook-Hyung Kim

South Korea

Reinhard Kirnbauer

Austria

Pravina Kitikoon

USA

Ronald Klein

USA

John Klena

USA

Regina Klimova

Russia

Jonas Klingström

Sweden

Jochen Klumpp

Switzerland

Barbara Klupp

Germany

Darwyn Kobasa

Canada

Michinori Kohara

Japan

Alain Kohl

UK

Satoshi Koike

Japan

Narcisse Patrice Komas

Central African Republic

Petr Kominek

Czech Republic

Gerrit Koopman

Netherlands

Neeltje Kootstra

Netherlands

Christian Körner

USA

Sergei Kosakovsky Pond

USA

Takumi Koshiba

Japan

Jill Koshiol

USA

Frederick Koster

USA

Shyam Kottilil

USA

Konstantin Kousoulas

USA

George Koutsoudakis

Spain

Philip Krause

USA

Tannette Krediet

Netherlands

Erna Geessien Kroon

Brazil

Andi Krumbholz

Germany

Claude Krummenacher

USA

Mart Krupovic

France

Ajit Kumar

USA

Sachin Kumar

India

Rajesh Kumar

India

Jiban Kundu

Czech Republic

Jane Kuypers

USA

Juan Kuznar

Chile

Chang-hee Kweon

South Korea

Paul Kwo

USA

Samuel Kojo Kwofie

South Africa

So Young Kwon

South Korea

Angie Lackenby

UK

Kelly Lager

USA

Ching Lung Lai

China

Ke Lan

China

Kamran Lankarani

Iran

Jason Lanman

USA

Lars Larsen

Denmark

Magdalena Larska

Poland

Jacques Le Pendu

France

Jason LeBlanc

Canada

Jin-Ching Lee

Taiwan

Kim Sung Lee

Singapore

Min-Shi Lee

Taiwan

Fan Lee

Taiwan

Jonathan Lee

Canada

Sang-Won Lee

Australia

Chang Lee

USA

David Lefebvre

Belgium

Elliot Lefkowitz

USA

Julian Leibowitz

USA

Petr Leiman

Switzerland

Porntippa Lekcharoensuk

Thailand

Guy Lemay

Canada

Elba Lemos

Brazil

Qibin Leng

China

Jean-Michel Lett

France

Ting Fan Leung

Hong Kong

Connie Leung

Hong Kong

Nicolas Lévêque

France

Laura Levy

USA

Penny Lewthwaite

UK

Yvan L’Homme

Canada

Jun Li

USA

Huixin Li

China

Kangsheng Li

China

Feng Li

USA

Sung-Chou Li

Taiwan

Huachun Li

China

Zejun Li

China

Mei-Ling Li

USA

Zheng-Lun Liang

China

Young-Suk Lim

South Korea

Thawornchai Limjindaporn

Thailand

Daniel Limonta

Cuba

Ming-Kuem Lin

Taiwan

Cheng-Wen Lin

Taiwan

Tong Lin

China

Tsun-Mei Lin

Taiwan

Debbie Lindell

Israel

Robbin Lindsay

Canada

Fang Liu

China

Ding Xiang Liu

Singapore

Bindong Liu

USA

Qinfang Liu

USA

Jixing Liu

China

Wenlan Liu

USA

Jinhua Liu

China

Hung-Jen Liu

Taiwan

Jiang Ning Liu

China

Wei Liu

China

Yuanyuan Liu

USA

Zhengwen Liu

China

Chen Liu

USA

Xiangtao Liu

China

Marcela Lizano

Mexico

Francisco Lobo

Brazil

Brian Long

USA

Yueh-Ming Loo

USA

Guillermo López

Spain

Xavier López

Spain

Marcelo Lopez-Lastra

Chile

Pierre Loulergue

France

Hongzhou Lu

China

Ling Lu

USA

Yinping Lu

China

John Ludbrook

Australia

Hanns Ludwig

Germany

Pam Dachung Luka

Nigeria

Alexander Lukashev

UK

Igor Lukashevich

USA

Francoise Lunel Fabiani

France

Min-Hua Luo

China

Xuejun Ma

China

Narender Maan

India

Beatrice Macchi

Italy

Michael Mach

Germany

Ian Mackay

Australia

Erich Mackow

USA

James Maclean

USA

Suresh Mahalingam

Australia

Ravi Mahalingam

USA

Shinji Makino

USA

Daniel Malamud

USA

Adriana Malheiro

Brazil

Viviana Malirat

Argentina

Alexander Malogolovkin

Russia

Carlos Maluquer de Motes

UK

Ioannis Mammas

Greece

Elisabetta Manaresi

Italy

Maria Jose Manata

Portugal

Niwat Maneekarn

Thailand

Nicholas Maness

USA

Kathy Mangold

USA

Satendra Kumar Mangrauthia

India

Balaji Manicassamy

USA

Annette Mankertz

Germany

Spilios Manolakopoulos

Greece

Shahid Mansoor

Pakistan

Qing Mao

China

Arnaud Marchant

Belgium

Philip Marcus

USA

Silvya Maria-Engler

Brazil

Vazeille Marie

France

Darren Patrick Martin

South Africa

Byron Martina

Netherlands

Isidoro Martinez

Spain

Liliana Amalia Martinez Peralta

Argentina

Encarna Martinez Salas

Spain

Jose Martinez-Costas

Spain

Luis Martinez-Sobrido

USA

Regina Martins

Brazil

Masaji Mase

Japan

Charles Masembe

Uganda

Tetsuro Matano

Japan

Anuja Mathew

USA

Shigenobu Matsuzaki

Japan

John McCauley

UK

John McKillen

UK

Áine McKnight

UK

Margaret McLaughlin-Drubin

USA

Bruce McManus

Canada

Louise-Anne McNutt

USA

Wilfried Meijer

Spain

José A. Melero

Spain

Francisco Mello

Brazil

Muhammad Ashraf Memon

Pakistan

Xiang-Jin Meng

USA

Zhongji Meng

China

Andreas Mentis

Greece

Ayalew Mergia

USA

Malik Merza

Sweden

William Messer

USA

Michael Metzger

USA

Florencia Meyer

USA

Andreas Meyerhans

Spain

Craig Meyers

USA

Scott Michael

USA

Peter Michielsen

Belgium

Victor Mikhailov

Russia

Eric Miller

USA

William Miller

USA

Craig Miller

USA

A Dusty Miller

USA

Vladimir Minin

USA

Tim Minogue

USA

Kiran Mir

USA

Daniel Miranda

Colombia

Ali Mirazimi

Sweden

John Mittler

USA

Tariq Moatter

Pakistan

Brenda Modak

Chile

Goudarz Molaei

USA

Sophie Molia

Mali

Debasis Mondal

USA

Isabella Monne

Italy

Stephan Monroe

USA

Jennifer Moodley

South Africa

Marc Morais

USA

Mariza Morgado

Brazil

Enrique Moriones

Spain

Hiromitsu Moriyama

Japan

Giulia Morsica

Italy

Alan Moss

USA

Michael Mossing

USA

Bruno Mota

Brazil

Morteza Motazakker

Iran

Christiane Mougin

France

Andrew J Mouland

Canada

Sylvester Rodgers Moyo

Namibia

Sunil Mukherjee

India

Mick Mulders

Luxembourg

Martin Muller

Germany

Hermann Muller

Germany

Egbert Mundt

Germany

Muhammad Munir

Sweden

Yoshiki Murakami

Japan

Yoshihiko Murata

USA

Eain Murphy

USA

Michael Murtaugh

USA

Joseph Mwangi

Kenya

Rafael Najera

Spain

Keiichiro Nakamura

Japan

Tatsunori Nakano

Japan

Hans Nauwynck

Belgium

David Navarro

Spain

Jesus Navas-Castillo

Spain

Muhammad Nawaz-ul-Rehman

Pakistan

Baibaswata Nayak

USA

Wilfred Ndifon

Israel

Francesco Negro

Switzerland

Martha Nelson

USA

Johan Neyts

Belgium

James Ng

USA

Lisa F.P. Ng

Singapore

Marie Nguyen

USA

Jack Nguyen

USA

Stuart Nichol

USA

John Nicholls

Hong Kong

Anthony Nicola

USA

Christian Niel

Brazil

Caroline Nilsson

Sweden

Ingo Nindl

Germany

Andreas Nitsche

Germany

Allan Nix

USA

Richard Njouom

Cameroon

Mauricio Nogueira

Brazil

Michael Nonnemacher

USA

Peter Norberg

Sweden

Helene Norder

Sweden

Elizabeth Norton

USA

Vlad Novitsky

USA

Sandro Filipe Nunes

UK

Ignacio Núñez

Spain

Patricia Nuttall

UK

M. Steven Oberste

USA

Jack Odle

USA

Tuba Cigdem Oguzoglu

Turkey

Tamaki Okabayashi

Thailand

Margaret Okomo-Adhiambo

USA

Gene Olinger

USA

Ledy Oliveira

Brazil

Victoria Olson

USA

Wieslawa Olszewska

UK

Tanja Opriessnig

USA

Massimo Origoni

Italy

Gérard Orth

France

Carla Osiowy

Canada

Pamela Österlund

Finland

Mario Ostrowski

Canada

David Ott

USA

Ilnour Ourmanov

USA

Ravindra P. Veeranna

USA

Kanti Pabbaraju

Canada

Slobodan Paessler

USA

Nicola Page

South Africa

Dasja Pajkrt

Netherlands

Narinder Pal

USA

Minna Paloniemi (née Risku)

Finland

Peter Palukaitis

South Korea

Ana Luiza Pamplona Mosimann

Brazil

Mark Pandori

USA

Ivona Pandrea

USA

Arturo Panduro

Mexico

Mary Pantin-Jackwood

USA

Ralph Pantophlet

Canada

George Papatheodoridis

Greece

Mark Parcells

USA

Manmohan Parida

India

Bongkyun Park

South Korea

Jong Park

South Korea

Alan Parker

UK

Scott Parker

USA

Francisco Parra

Spain

Deborah Parris

USA

Colin Parrish

USA

John Pasick

Canada

Basavaprabhu Patil

Switzerland

B. Pattnaik

India

John Patton

USA

Sujay Paul

India

Christopher Payan

France

John Pearce

USA

Pedro Pedrosa

Brazil

Lee Peng

USA

Krisana Pengsaa

Thailand

Beatriz Perdiguero

Spain

María Dolores Pérez

Spain

Robert Perni

USA

Alessandro Perrella

Italy

Hervé Perron

Switzerland

Boris Matija Peterlin

USA

Michel Peterschmitt

France

Tung Phan

USA

Antonio J Piantino Ferreira

Brazil

Andreas Pichlmair

Germany

Alessandra Pierangeli

Italy

Sylvie Pillet

France

Qian Ping

China

Aguinaldo Pinto

Brazil

Rosa Maria Pintó

Spain

Giulio Pisani

Italy

Massimo Pizzato

Italy

Hidde Ploegh

USA

Sébastien Plumet

France

Jane Polston

USA

Malgorzata Pomorska-Mol

Poland

Raffaello Pompei

Canada

Art Poon

Canada

Yong Poovorawan

Thailand

Mahmoudreza Pourkarim

Belgium

Penny Powell

UK

Bruno Pozzetto

France

Ramesh Prabhu

USA

Afiono Agung Prasetyo

Indonesia

Jan Pravsgaard Christensen

Denmark

María Victoria Preciado

Argentina

Mark Prichard

USA

Jose A Prieto

Spain

Jose Luiz Proenca-Modena

Brazil

Maria Carmen Puertas

Spain

Carlos Alberto Pujol

Argentina

Jennifer Pullium

USA

Pilaipan Puthavathana

Thailand

Krzysztof Pyrc

Poland

Zhikang Qian

China

Li Qihan

China

Zhou Ming Qin

China

Aijian Qin

China

Hua-Ji Qiu

China

Jorge Quarleri

Argentina

Raul Rabadan

USA

Holger F. Rabenau

Germany

Sonia Mara Raboni

Brazil

Paula Rahal

Brazil

Angamuthu Raja

India

Ramaswamy Raju

USA

Amitis Ramezani

Iran

Maria Rapicetta

Italy

Giovanna Rappocciolo

USA

Maria Raptopoulou-Gigi

Greece

Luca Rastrelli

Italy

Parntep Ratanakorn

Thailand

Ana Paula Ravazzolo

Brazil

Nikolai Ravin

Russia

K. Raviprakash

USA

Stuart Ray

USA

Naidu Rayapati

USA

Alfredo Rebora

Italy

Stewart Reid

Zambia

Rolf Renne

USA

Gourapura Renukaradhya

USA

Silvia Restrepo

Colombia

Miriam Reuschenbach

Germany

Peter Revill

Australia

Julio Reyes-Leyva

Mexico

Andrew Rice

USA

Juergen Richt

USA

Guus Rimmelzwaan

Netherlands

Felix C. Ringshausen

Germany

Maria Rios

USA

Guillermo Risatti

USA

Antonio Riva

UK

Michael Robek

USA

Kim Roberts

Ireland

Shelly Robertson

USA

Joan Robinson

Canada

Barry Rockx

USA

Belen Rodriguez

Spain

Peter Roeder

UK

Paulo Roehe

Brazil

John Roehrig

USA

Olivier Rohr

France

Bernard Roizman

USA

Michael Roner

USA

Lijun Rong

USA

Rachel Roper

USA

Pierre Roques

France

Karyna Rosario

USA

Timothy Rose

USA

Robert Rose

USA

Lisa Ross

USA

Paul Rota

USA

Xavier Roucou

Canada

Adib Rowhani

USA

Dirk Roymans

Belgium

Luis Rubio

Spain

Nicolas Ruggli

Switzerland

Till Rümenapf

Germany

Mariagrazia Rumi

Italy

Emily Rumschlag-Booms

USA

Wu Run

China

Sarah Russell

Australia

Andrew Rutenberg

Canada

Kate Ryman

USA

Wi-Sun Ryu

South Korea

David Safronetz

USA

Bruno Sainz

Spain

Takafumi Saito

Japan

Mika Saito

Japan

Juan-Carlos Saiz

Spain

Margarita Sáiz

Spain

Yoshihiro Sakoda

Japan

Toshie Sakuma

USA

Romina Salpini

Italy

Maria Salvato

USA

I-Ching Sam

Malaysia

Dhanraj Samuel

UK

Sabri Sanabani

Brazil

Gloria Sanchez Moragas

Spain

Yongming Sang

USA

Daniel Santos Mansur

Brazil

Bruno Sargueil

France

Shiv Sarin

India

Vijaya Satchidanandam

India

Christian Sauder

USA

Andrea Savarino

Italy

Giovanni Savini

Italy

Murat Sayan

Turkey

David Schaffer

USA

Dena Schanzer

Canada

Joerg Schenk

Germany

Leonardo Schiavon

Brazil

Oliver Schildgen

Germany

John Schiller

USA

Scott Schmid

USA

Paul Schnitzler

Germany

Karen-Beth Scholthof

USA

Declan Schroeder

UK

Ulrich Schubert

Germany

Ronald Schultz

USA

Stacey Schultz-Cherry

USA

Linda Scobie

UK

Eduardo Scodeller

Argentina

Christoph Seeger

USA

Michel Segondy

France

Ulrike Seifert

Germany

Ulrike Seitzer

Germany

Hossein Sendi

USA

Donald Senear

USA

Sharmila Sengupta

India

Muhieddine Seoud

Lebanon

Ethan Settembre

USA

Alberto Severini

Canada

David Severson

USA

Melkote Shaila

India

Madhusudana Shampur

India

Hong Shang

China

Wei Shao

USA

Elizabeth Sharlow

USA

Bhaskar Sharma

India

Pradeep Sharma

India

Hitt Sharma

India

Huigang Shen

USA

Dionne Shepherd

South Africa

Zhongjie Shi

USA

Huoying Shi

China

Kyoko Shinya

Japan

Bruce Shiramizu

USA

Sujan Shresta

USA

Deepak Shukla

USA

Joanna Siennicka

Poland

Wong Siew Tung

Malaysia

Kumud Singh

USA

Raj Kumar Singh

India

Tim Skern

Austria

Nicolas Sluis-Cremer

USA

Donald Smee

USA

Jolanda Smit

Netherlands

Duncan R. Smith

Thailand

Janet Smith

USA

Heidi Smuts

South Africa

Francisco Sobrino

Spain

Hooreieh Soleimanjahi

Iran

Alicia Solorzano

USA

Changxu Song

China

Elijah Songok

Kenya

Sieghart Sopper

Austria

Stanislav Sosnovtsev

USA

George Sourvinos

Greece

Erica Spackman

USA

Ellen Sparger

USA

Maria Spies

USA

Christina Spiropoulou

USA

E. Sreekumar

India

Villuppanoor Alwar Srinivasan

India

Arun Srivastava

USA

Karl Stahl

Sweden

Anthony Staines

Ireland

Danica Stanekova

Slovakia

James Stanton

USA

Mariane Stefani

Brazil

Eike Steinmann

Germany

Edward Stephens

USA

Joanna Stewart

New Zealand

Andrej Steyer

Slovenia

Cristina Stoyanov

USA

G. Thomas Strickland

USA

Alex Strongin

USA

Elankumaran Subbiah

USA

Makoto Sugiyama

Japan

Amorsolo Suguitan

USA

Rebecca Sumner

UK

Zhi Sun

USA

Shulei Sun

USA

Sujatha Sunil

India

Vijay Suppiah

Australia

Camille Sureau

France

Leonardo Susta

USA

Gerd Sutter

Germany

Fumitaka Suzuki

Japan

Valentina Svicher

Italy

Jozsef Szeberenyi

Hungary

Ashraf Tabll

Egypt

Oscar Taboga

Argentina

Gilda Tachedjian

Australia

Fumihiro Taguchi

Japan

Shigeru Tajima

Japan

Ayato Takada

Japan

Kazuaki Takehara

Japan

Yasuhiro Takeuchi

UK

Laura Talarico

Argentina

Michael Talledo

Peru

Wenjie Tan

China

Eng Lee Tan

Singapore

Junko Tanaka

Japan

Yasuhito Tanaka

Japan

Yi-Wei Tang

USA

Qinghai Tang

China

Yasunori Tanji

Japan

Jayanta Tarafdar

India

Bogdan Tarus

France

Roongroje Thanawongnuwech

Thailand

Niroshan Thanthrige-Don

Canada

Chutima Thepparit

Thailand

Yves Thomas

Switzerland

Kegong Tian

China

Zhijun Tian

China

Peter Tijssen

Canada

Laurence Tiley

UK

B. Karsten Tischer

Germany

Kelvin To

Hong Kong

Anna Tomas

Spain

Stephen Mark Tompkins

USA

Huahua Tong

USA

Dewen Tong

China

Guang-Zhi Tong

China

Ivan Toplak

Slovenia

Montserrat Torremorell

USA

Carolina Torres

Argentina

Carlo Torti

Italy

Sodsai Tovanabutra

USA

Jonathan Towner

USA

Rita Trammell

USA

Nirupma Trehanpati

India

Marten Trendelenburg

Switzerland

Joanne Trgovcich

USA

Benjamin Trible

USA

Vladimir Trifonov

USA

Nagesh Tripathi

India

Ralph Tripp

USA

Edward Trybala

Sweden

Ching-Hsiu Tsai

Taiwan

Herman Tse

Hong Kong

Boldtsetseg Tserenpuntsag

USA

Konstantin Tsetsarkin

USA

Kyoko Tsukiyama-Kohara

Japan

Loretta Tuosto

Italy

Patricia Turner

Canada

Jean-Claude Twizere

Belgium

Sukathida Ubol

Thailand

Kenichi Umene

Japan

Hoshang Unwalla

USA

Susan Uprichard

USA

Andi Utama

Indonesia

Astrid Vabret

France

Iris Valdes Prado

Cuba

Steven Valles

USA

Steven Van Borm

Belgium

Gerlinde van de Walle

Belgium

Rafael van den Bergh

Belgium

Lia van der Hoek

Netherlands

Tonja van der Kuyl

Netherlands

Wim van der Poel

Netherlands

Sabine van der Sanden

USA

Anne van Diepeningen

Netherlands

James van Etten

USA

Marit van Gils

Netherlands

Daniel van Langenberg

Australia

Carine van Lint

Belgium

Debby van Riel

Netherlands

Vicky van Santen

USA

Eva Vareckova

Slovakia

Michael Veit

Germany

Ramamurthy Venkataramanan

India

Gyorgy Veress

Hungary

Helene Verheije

Netherlands

Massimiliano Veroux

Italy

Matias Victoria

Uruguay

Pierre-Olivier Vidalain

France

Mauro Viganò

Italy

Paluru Vijayachari

India

Dhanasekaran Vijaykrishna

Singapore

Stefan Vilcek

Slovakia

Francois Villinger

USA

Muwanika Vincent

Uganda

Amy Vincent

USA

Reinhard Vlasak

Austria

Antje Voigt

Germany

Sudhanshu Vrati

India

Wahala Wahala

USA

Yasir Waheed

Pakistan

Diane Waku-Kouomou

USA

Peter Walker

Australia

Guoqiang Wan

USA

Xiu-Feng (Henry) Wan

USA

Zhiliang Wang

China

Qinghua Wang

USA

David Wang

USA

Hongning Wang

China

Wenbing Wang

China

Qian Wang

USA

Wei-Kung Wang

USA

Xifeng Wang

China

Scott Weaver

USA

Richard Webby

USA

E. Scott Weber III

USA

Robert Webster

USA

Hana Weingartl

Canada

Christoph Welsch

Germany

Johan Westin

Sweden

James Whitehorn

Viet Nam

Ulrike Wieland

Germany

Christer R. Wiik-Nielsen

Norway

Hermann Willems

Germany

Anna-Lise Williamson

South Africa

Jan Wilschut

Netherlands

Carolyn Wilson

USA

Kim Wilson

Australia

Michael Winkler

Germany

Christiane Wobus

USA

Roman Wölfel

Germany

Frank Wong

Australia

Yuntao Wu

USA

Hongzhuan Wu

USA

Carol Wyatt

USA

Harry Xia

USA

Yan Xiang

USA

Zhengguo Xiao

USA

Hang Xie

USA

Youhua Xie

China

Yan Xie

China

Kemin Xu

USA

Dongping Xu

China

Wenbo XU

China

Ming Xu

China

Teruo Yamashita

Japan

Huimin Yan

China

Tohru Yanase

Japan

Jibing Yang

USA

Hanchun Yang

China

Wei Yang

China

Yoshihiko Yano

Japan

Lunguang Yao

China

Feng Yao

USA

Junichiro Yasunaga

Japan

Belinda Yen-Lieberman

USA

Jonathan Yewdell

USA

Wang Yin

China

Lu Yongzhong

China

Dongwan Yoo

USA

Nobuyuki Yoshikawa

Japan

Ying Yu

China

Fan Yuchen

China

Thomas Yuill

USA

Cihan Yurdaydin

Turks and Caicos Islands

Zichria Zakay-Rones

Israel

Alexander Zakhartchouk

Canada

Michele Zampieri

Italy

Hassan Zaraket

USA

Roland Zell

Germany

F. Murilo Zerbini

Brazil

Bo Zhang

China

Zhidong Zhang

Canada

Xin-Xin Zhang

China

Jennifer Zhang

USA

Yanjin Zhang

USA

Gui-Hong Zhang

China

Chuan-Xi Zhang

China

Keshan Zhang

China

Jiangqin Zhao

USA

Jianjun Zhao

China

Chunfang Zheng

Canada

Shusen Zheng

China

Tao Zheng

New Zealand

Ming-Hua Zheng

China

Oleg Zhirnov

Russia

Ping Zhong

China

Yanwei Zhong

China

Rong Zhou

China

Jiyong Zhou

China

Yusen Zhou

China

Guohui Zhou

China

Yumei Zhou

USA

Bin Zhou

USA

Guoqiang Zhu

China

Fanxiu Zhu

USA

Haizhen Zhu

China

Snjezana Zidovec Lepej

Croatia

Laszlo Zsak

USA

